# Biochemical properties of pancreatic colipase from the common stingray Dasyatis pastinaca

**DOI:** 10.1186/1476-511X-10-69

**Published:** 2011-05-08

**Authors:** Abir Ben Bacha, Aida Karray, Lobna Daoud, Emna Bouchaala, Madiha Bou Ali, Youssef Gargouri, Yassine Ben Ali

**Affiliations:** 1 Laboratoire de Biochimie et de Génie Enzymatique des Lipases, ENIS route de Soukra, BP1173, University of Sfax - 3038 Sfax, Tunisia

## Abstract

**Background**

Pancreatic colipase is a required co-factor for pancreatic lipase, being necessary for its activity during hydrolysis of dietary triglycerides in the presence of bile salts. In the intestine, colipase is cleaved from a precursor molecule, procolipase, through the action of trypsin. This cleavage yields a peptide called enterostatin knoswn, being produced in equimolar proportions to colipase.

**Results**

In this study, colipase from the common stingray *Dasyatis pastinaca *(CoSPL) was purified to homogeneity. The purified colipase is not glycosylated and has an apparent molecular mass of around 10 kDa. The NH_2_-terminal sequencing of purified CoSPL exhibits more than 55% identity with those of mammalian, bird or marine colipases. CoSPL was found to be less effective activator of bird and mammal pancreatic lipases than for the lipase from the same specie. The apparent dissociation constant (Kd) of the colipase/lipase complex and the apparent Vmax of the colipase-activated lipase values were deduced from the linear curves of the Scatchard plots. We concluded that Stingray Pancreatic Lipase (SPL) has higher ability to interact with colipase from the same species than with the mammal or bird ones.

**Conclusion**

The fact that colipase is a universal lipase cofactor might thus be explained by a conservation of the colipase-lipase interaction site. The results obtained in the study may improve our knowledge of marine lipase/colipase.

## Introduction

Lipases (triacylglyceride ester hydrolase, EC 3.1.1.3) catalyze the hydrolysis of triglycerides at the lipid-water interface. In contrast to esterases which exhibit their maximal activity on soluble substrate, lipases require the presence of an interface to be fully active [1]. It is established that the accumulation of amphiphiles at the oil/water interface in the duodenum of vertebrates (the oil-in-water droplets are covered by several natural surfactants) prevent pancreatic lipase (PL) binding.

Pancreatic colipase is a required co-factor for pancreatic lipase, being necessary for its activity during hydrolysis of dietary triglycerides in the presence of bile salts [2]. In the intestine, colipase is cleaved from a precursor molecule, procolipase, through the action of trypsin [2]. This cleavage yields a peptide called pentapeptide called enterostatin knoswn to regulate food intake in higher mammals, being produced in equimolar proportions to colipase [3,4].

The understanding of PL activation and catalysis has progressed dramatically thanks to the determination of the three-dimensional structures of the uncomplexed human PL by Winkler et al. [5] and of a human pancreatic lipase/porcine colipase complex studied in the presence of mixed micelles by van Tilbeurgh et al. [6]. In the uncomplexed enzyme, a large amphiphilic loop (the flap) blocks the active site access, thus explaining the limited catalytic activity of pancreatic lipase in solution. The pancreatic lipase/porcine colipase complex [6], in turn, has the active site exposed and the flap establishes several polar contacts with colipase. This rearrangement gives rise to an extensive hydrophobic surface [6], which may be involved in the interaction of the pancreatic lipase/colipase complex with tri- and diglyceride substrate.

Later, Hermoso et al., (1996) have published the crystal structure of the porcine pancreatic lipase/porcine colipase complex from crystals obtained using the non-ionic detergent tetraethylene glycol monooctyl ether (C_8_E_4_) and they found that PLhad the open conformation and attributed this result to the presence of detergent micelles in the crystallization medium. In fact, they have also observed that inhibition of PL by the serine-specific inhibitor diethyl *p*-nitrophenylphosphate (E600) in solution, a reaction that requires an accessible active site, only takes place in the presence of pancreatic lipase, colipase and supermicellar concentrations of either non-ionic detergents or bile-salts [7].

The biology and the biochemistry of mammalian colipase are well documented and several studies have provided evidence that no difference can be observed among mammals in the activation of a PL from one species by colipase from another species when emulsified triolein or tributyrin (TC4) is used as a substrate [8,9]. However, a recent study showed that bird and mammal lipases are more activated by their own colipases. This finding can be attributable to a higher specificity of the colipase-lipase interaction [10].

The aquatic world contains a wide variety of living species and represents a great potential for discovering new proteins. However, in our knowledge, no colipase from the marine vertebrate have been purified so far except for the *Squalus acanthius *which was the first colipase purified and characterized from the dogfish pancreas [11].

It is therefore interesting to study some catalytic and biochemical properties of another purified marine colipase to gain more information about the marine pancreatic colipase function. This paper reports the purification to homogeneity of an active pancreatic colipase from the common stingray *Dasyatis pastinaca*. This colipase, tentatively named stingray pancreatic colipase (CoSPL) was characterized with respect to its biochemical properties.

## Results and discussion

### Purification of CoSPL and NH_2_-terminal sequence determination

CoSPA was purified according to the procedure described in materials and methods. The purification flow sheet is summarized in Table 1. After the Mono Q chromatography, the purification factor reached 56 fold with a high recovery yield of 35% of the initial colipase activity. The specific activity of the purified CoSPL was found to be 9200 U/mg when olive oil emulsion was used as a substrate at pH 8.5 and 37°C and in the presence of 6 mM NaTDC.

**Table 1:** Flow sheet of stingray pancreatic colipase purification

Purification step	**Total**^***(a) ***^**activity (units)**	**Protein**^***(b) ***^**(mg)**	Specific activity (U/mg)	Activity recovery (%)	Purification factor
**Heat and acidic treatment**	78000	500	156	100	1

**(NH**_**4**_**)**_**2**_**SO**_**4 **_**Precipitation (50%)**	62400	200	312	80	2

**Ethanol fractionation (50-90%)**	48672	50	973.4	62.4	6.24

**Mono S Sepharose**	36500	15.5	2354.8	46.8	15.1

**Mono Q Sepharose**	27400	2.98	9200	35.1	59

^*(a) *^1 Unit: One colipase unit corresponds to the amount of the cofactor that increases bile salts inhibited pancreatic lipase activity by one enzyme unit.

^*(b) *^Proteins were estimated by Bradford method [24]. The experiments were conducted three times.

**Figure 1:**
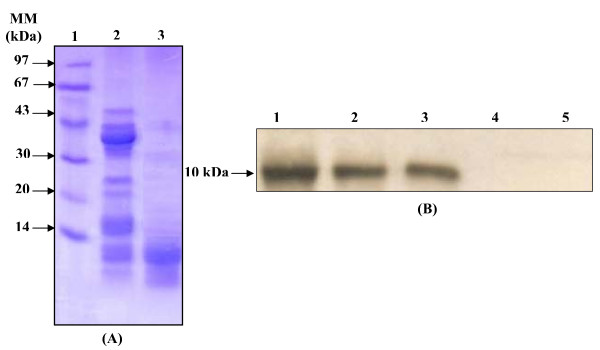
**SDS-PAGE (15%) and Immunoblot analysis of CoSPL**. (A) Analysis of purified of pure CoSPL by SDS-PAGE (15%). Lane 1, molecular mass markers (Pharmacia); Lane 2, CoSPL solution (20 μg) obtained after Mono S chromatography; lane 3, purified CoSPL (15 μg) obtained after Mono Q chromatography. The gel was stained with Coomassie blue. (B) Immunoblot analysis, pure CoOPL (15 μg) (lane 1), CoTPL (15 μg) (lane 2), CoCPL (15 μg) (lane 3), CoDrPL (30 μg ) and CoSPL (30 μg) (lane 5) using anti-CoOPL serum at 1:500 dilution.

After the anion exchange chromatography, the fractions containing CoSPL were pooled and analyzed on SDS-PAGE. Figure 1A shows that CoSPL has an apparent molecular mass of around 10 kDa. This value is in agreement with the molecular mass estimated using a gel filtration Superose 12 column by fast-protein liquid chromatography (data not shown).

Altogether, these results suggest that marine colipase is a monomeric protein, as discribed for mammal and bird colipases.

The NH_2_-terminal sequencing of purified CoSPL allowed the unambiguous identification of 25 residues. The alignment of CoSPL sequence with those of dogfish [11], chicken [12], turkey [10], porcine [2,13], and dromedary [10] pancreatic colipases is shown in Table 2. CoSPL NH_2_-terminal sequence exhibits more than 55% identity with those of mammalian, bird or marine colipases (Table 2).

**Table 2:** Alignment of the N-terminal sequence of Stingray colipase with dog fish, turkey, dromedary, chicken and pig pancreatic colipases

		**6**	**12**	**18**	**24**	**30**	**36**	
Stingray		GIFLNL	SAGEIC	IGSFQC	KSSCCQ	RETGLS	LAR	[This study]

Dog fish		GLFLNL	SAGEIC	VGSFQC	KSSCCQ	HETGLS	LAR	[11]

Ostrich		GLVFNL	ETGELC	LQSAQC	RSHCCH	RSDGLS	LAR	[10]

Turkey		GLIFNL	DTGELC	VQSAQC	QSGCCQ	YDSGLS	LAR	[10]

Dromedary		GIVINL	DTGELC	LNSAQC	RSHCCH	RADGLS	LAR	[10]

Chicken		GLIFNL	DTGELC	LQSAQC	KSECCQ	EDSGLS	LAX	[22]

Pig	VPDPR	GIIINL	DEGELC	LNSAQC	KSNCCQ	HDTILS	LSR	[2,14]

Humain		GIIINL	DEGELC	LNSAQC	KSNCCQ	HDTILS	LLR	[6]

rat		GLFINL	EDGEIC	VNSMQC	KSRCCQ	HDTILG	IAR	[4]

For comparison, residues in bold indicate identical amino acids.

The presence of eventual glycan chains in pure colipase molecule was investigated. Our results showed that the purified protein is not glycosylated (data not shown).

### Effect of colipase on the tributyrin hydrolysis rate by pancreatic lipase

It has been established that some mammalian pancreatic lipases lack enzymatic activity when TC_4 _is used as substrate in the absence of bile salts and colipase. The high energy existing at the tributyrin/water interface could be responsible for their irreversible denaturation [14].

**Figure 2:**
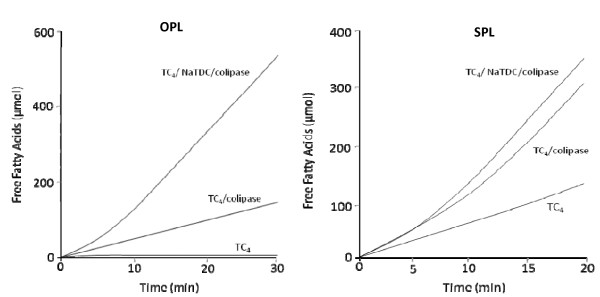
**Comparison of same kinetic properties of OPL and SPL**. (A) Kinetic of hydrolysis of tributyrin emulsion by OPL (22 U) [10]. (B) Kinetic of hydrolysis of tributyrin emulsion by SPL (22 U). Lipolytic activity was followed at pH 8.5 and 37°C in the absence or in the presence of a molar excess of ostrich pancreatic colipase = 5 and 4 mM NaTDC.

In contrast to ostrich pancreatic lipase (OPL) (figure 2A), which failed to catalyze the hydrolysis of triacylglycerols at high interfacial energy (TC_4_) [10], SPL is able to hydrolyse efficiently the pure TC_4 _(figure 2B). When colipase was added to the lipolytic system, OPL was protected against surface denaturation. Nevertheless, cofactors cannot totally protect enzymes from interfacial inactivation. The combined effect of colipase and bile salts is most effective in preventing this denaturation (figure 2). The curve representative of the hydrolysis rate of TC_4 _remained linear more than 15 min when bile salts and colipase were added together prior to the lipase in the lipolytic medium independly of the colipase origin (figure 2). Our findings confirm the idea that in the presence of bile salts, colipase helps to keep lipase at the interface at high energy and linearises its kinetics.

### Activation of bile salts inhibited stingray lipase by colipases from various species

It is well established that bile salts are strong inhibitors of all pancreatic lipases independently of their origins [14,15]. At low concentration (below CMC) bile salts stabilize lipase at interfaces [16]. Neverless, higher concentration of bile salt inhibit the lipase activity by desorbing it from its substrate (interface).

A previous comparative study showed that mammalian colipase presents lower activation effect towards bird lipases than the bird counterpart [10]. In order to get more information about the pancreatic lipase-colipase complex function specificity, we investigated, in this study, the activation of the bile salt-inhibited SPL by pure colipase from dromedary, turkey, chicken and stingray. Activity of pure PLagainst emulsified olive oil was determined at increasing concentrations of bile salts in the absence or in the presence of a molar excess of colipase (data not shown). NaTDC was shown to act as a strong inhibitor of pancreatic lipase. Inhibition was reversed after addition of stingray colipase to the assay system. No significant difference was observed regarding the ability of pure colipase from dromedary, chicken or turkey to activate bile-salt-inhibited SPL (data not shown).

**Figure 3:**
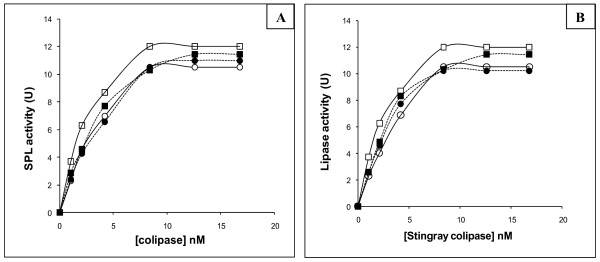
**the activation of SPL by colipases from other species and the capacity of CoSPL to activate various pancreatic lipases**. (A) Effect of varying amounts of stingray, dromedary, turkey and chicken pancreatic colipases on the activity of pure SPL in the presence bile salts at pH 8.5 and 37°C. Enzymatic activity of lipase (2.1 nM) was assayed in the presence of 6 mM NaTDC with increasing amounts of colipase. (open circle): CoCPL; (open square): CoDrPL; (black square): CoSPL; (black circle): CoTPL (B) Effect of varying amounts of stingray pancreatic colipase on the activity of pure lipases from various species in the presence bile salts at pH 8.5 and 37°C. Enzymatic activity of lipase (2.1 nM) was assayed in the presence of 6 mM NaTDC with increasing amounts of colipase. (open circle): CoCPL; (open square): CoDrPL; (black square): CoSPL; (black circle): CoTPL

To check the affinity between SPL and colipases isolated from different species, enzymatic activity was measured using emulsified olive oil as substrate under standard conditions in the presence of 6 mM NaTDC at increasing concentrations of purified colipase from stingray, chicken, turkey, and dromedary tissue (figure 3A). Under our experimental conditions, the maximal lipase activity was obtained with a molar ratio lipase/colipase of about 1:2. SPL was found to be activated by all pure pancreatic colipases tested, independently of their origins. However, we noted that mammal and bird colipases were less effective activators of marine enzyme than the marine cofactor (figure 3A). In the same direction, Figure 3B showed that CoSPL was found to be less effective activator of bird and mammal enzymes than for its homologous lipase.

To determine the apparent dissociation constant (Kd) of lipase-colipase complex, the linear curves corresponding to the Scatchard representation were plotted (data not shown) based on the results of figure 3 as described by Rathelot et al. [17]. The Kd values of different lipase-colipase complex were determined from the slope of the linear curves of the Scatchard plots. The apparent Vmax values were also deduced from figure 3. Then, the ratio representing the catalytic efficiency (kcat/Kd) was calculated for SPL (Table 3) and for bird and mammal lipases as well (table 4). From these values, it can be concluded that the ratio kcat/Kd between CoSPL and SPL is higher (2073) than that between CoSPL and bird or mammal lipases (the ratio kcat/Kd varying from 611 to 930) as well as between SPL and bird or mammal colipases (the ratio kcat/Kd varying from 599 to 2073).

**Table 3:** Kinetic parameters between stingray lipase and colipases of various species deduced from figure 4A

	CoSPL	CoTPL	CoCPL	CoDrPL
**Apparent Kd (10**^**-9 **^**M)**	1.2 ± 0.01	3.6 ± 0.2	3.9 ± 0.31	2.2 ± 0.15

**Apparent Vmax (μmol min**^**-1**^**mg**^**-1**^**)**	3200 ± 120	2895 ± 150	2799 ± 120	3047 ± 225

**kcat (S**^**-1**^**)**	2400 ± 156	2171 ± 96	2242 ± 154	2539 ± 213

**kcat/Kd (S**^**-1**^**M**^**-1 **^**10**^**-9**^**)**	2073 ± 95	599 ± 32	576 ± 36	1137 ± 160

The apparent Kd were deduced from the slope of the linear curves of the Scatchard plots. The experiments were conducted three times.

**Table 4:** Kinetic parameters between stingray colipase and lipases of various species deduced from figure 4B

	**apparent Kd (10**^**-9 **^**M)**	**apparent Vmax (μmol min**^**-1**^**mg**^**-1**^**)**	**kcat (S**^**-1**^**)**	**kcat/Kd (S**^**-1**^**M**^**-1 **^**10**^**-9**^**)**
**SPL**	1.15 ± 0.05	3200 ± 102	2400 ± 95	2074 ± 107

**TPL**	2.9 ± 0.1	3655 ± 85	2741 ± 150	946 ± 48

**CPL**	4.5 ± 0.5	3428 ± 132	2747 ± 87	611 ± 30

**DrPL**	3 ± 0.2	3341 ± 135	2785 ± 135	930 ± 71

The apparent Kd were deduced from the slope of the linear curves of the Scatchard plots. The experiments were conducted three times.

This might reflect the higher ability of the marine enzyme to interact with colipase from the same species than with the mammal or bird ones when olive oil was used as substrate. This result might suggest that this difference in the affinity towards pancreatic lipases from different species might be related to the structural differences between the marine, bird and the mammal colipases.

Previous works established the fact that cofactors from different mammal species are interchangeable and fully activate mammal pancreatic lipases [18-20]. The same value of Kd (1.1 10^-9 ^M) was obtained by Rathelot et al. when horse lipase was used, in presence of substrate, to form a complex with colipases from horse, pig or ox [17]. Recently it has been shown that bird and mammal lipases are more activated by their own colipases [10].

### Immunoblot analysis

Western blotting experiment was performed to check the immunological cross-reactivity of colipases from different species with anti-CoOPL serum. Only bird colipases reacted strongly with anti CoOPL (Figure. 1B). No cross-immunoreactivity was detected between anti-CoOPL serum and stingray or dromedary colipases. This might be explained by the fact that CoSPL surface exposed antigenic determinants are different from those present on bird pancreatic colipase surfaces.

Although the 32 residues of CoSPL NH_2_-terminal end showed significant homology with those of bird and mammal pancreatic colipases, the absence of immunoreactivity between CoSPL and anti-CoOPL serum strengthen the hypothesis that the amino acid exposed on the CoSPL surface, which are the main antigenic determinant, could be different from those of bird pancreatic colipase. This hypothesis needs further structural and biochemical investigations. Work is on progress in our laboratory to investigate this hypothesis.

## Materials and methods

### Chemicals

Tributyrin (99%, puriss) and benzamidine were from Fluka (Buchs, Switzerland), Triton X-100, β-mercaptoethanol (βME), bovine serum albumin (BSA) (99%, puriss), sodium taurodeoxycholate (NaTDC) was from Sigma Chemical (St. Louis, USA) and nitrocellulose membrane were from Sigma Chemical (St. Louis, USA), arabic gum was from Mayaud (Dagenham, UK), acrylamide and bis-acrylamide electrophoresis grade were from Bio-Rad (Paris, France), marker proteins and supports of chromatography used for CoSPL purification, Mono-Q Sepharose, Mono-S Sepharose were from Pharmacia (Uppsala, Sweden), PVDF membrane and protein sequencer Procise 492cLC provided from Applied Biosystems (Roissy, France), pH-stat was from Metrohm (Herisau, Switzerland).

### Pancreas collections

Stingrays (*Dasyatis pastinaca*) pancreases were collected from a local fish market (Sfax, Tunisia) and stored at -20°C.

### Enzymes and proteins

Dromedary, turkey and chicken pancreatic lipases were prepared in our laboratory as described previously [21]. Stingray pancreatic lipases was purified in our laboratory (unpublished data). Dromedary, turkey and chicken pancreatic colipases were purified according to previous works [10,12].

### Delipidation of pancreases

After decongelation, pancreases were cut into small pieces (1-2 cm^2^) and delipidated according to the method described previously [22]. After delipidation, about 15 g of delipidated powder of each pancreas were obtained from 100 g of fresh tissue.

### Determination of lipase and colipase activities

Lipase activity was measured titrimetrically at pH 8.5 and 37°C with a pH-stat, under the standard assay conditions described previously, using olive oil emulsion [18] or TC_4 _(0.25 ml) in 30 ml of 2.5 mM Tris-HCl and 1 mM CaCl_2_, pH 8.5 [23], as substrate. Some lipase assays were performed in the presence or absence of NaTDC and colipase. One lipase unit corresponds to 1 μmol of fatty acid liberated per minute.

Colipase activity was measured at pH 8.5 and 37°C as described by Rathelot et al. [23]. One colipase unit corresponds to the amount of cofactor that increases bile salt-inhibited PLactivity by 1 enzyme unit.

### Determination of protein concentration

Protein concentration was determined as described previously [24], using bovine serum albumin (E^1%^_1 cm _= 6.7) as reference.

### Oligosaccharide content

The presence of glycan chains in the purified cofactors was checked by the anthrone-sulfuric acid method using glucose as a standard [25].

### Alkylation of Cys residues

The alkylation of Cys residues of colipase was performed using the technique described by Okazaki, Yamada, and Imoto [26]. One milligram of cofactor in 1 ml of 10 mM Tris-HCl and 10 mM NaCl, pH 8.2, was denaturated in 375 μl of 8 M guanidine hydrochloride, 125 μl of 1 M Tris-HCl, 4 mM EDTA, pH 8.5, and 80 mM DTT during 30 min at 60°C. *S*-Pyridylethylation of cysteine residues of protein was performed by adding 4 μl of vinyl pyridine during 3 h at 25°C. The modified colipase was dialyzed against water for N-terminal sequencing.

### SDS-PAGE and immunoblotting technique

Analytical polyacrylamide gel electrophoresis of proteins in the presence of sodium dodecyl sulfate (SDS-PAGE) was performed following the method of Laemmli [27]. The proteins were stained either with Comassie brilliant blue or silver nitrate. Samples for sequencing were electroblotted according to Bergman and Jörnvall [13]. Protein transfer was performed during 1 h at 1 mA/cm^2 ^at room temperature.

The reactivity of anti-CoOPL serum with CoSPL, CoOPL, CoTPL and CoDrPL was checked using immunoblotting technique. After protein transfer, membranes were rinsed three times with PBS (phosphate buffer saline: 10 mM phosphate pH 7.2, 150 mM NaCl), then saturated with 3% of milk powder in PBS (saturating buffer) for 1 h at room temperature. Thereafter, anti-CoOPL serum diluted at 1:500 with PBS containing 0.05% Tween-20 (PBS/Tween-20) was incubated with the membranes for 1 h at room temperature. Afterwards, membranes were washed three times with PBS/Tween-20 then incubated for 1 h at room temperature with a 1:2000 dilution of alkaline phosphatase-conjugated anti-rabbit immunoglobulin (Sigma). After washing as mentioned above, membranes were incubated with a phosphatase substrate solution containing 0.3 mg/ml of nitroblue tetrazolium chloride (Sigma), 0.2 mg/ml of 5-bromo-4-chloro-3 indolyl-phosphate (Sigma) and 0.2 mg/ml of MgCl_2 _to reveal the specific immunoreactivity.

### NH_2_-terminal sequence analysis

The NH_2_-terminal end of ray pancreatic colipase was sequenced by automated Edman's degradation using an Applied Biosystems Protein sequencer Procise 492 cLC [28].

### Purification of pancreatic colipase from the stingray pancreas

Ten grams of stingray pancreas delipidated powder were suspended in 150 ml of water containing 2 mM benzamidine, 150 mM NaCl, and 0.2% Triton X-100 (v/v) and ground mechanically twice for 30 s at 4°C using the Waring Blendor system. The mixture was stirred with a magnetic bar for 30 min at 4°C and then centrifuged for 30 min at 12,000 rpm.

#### - Heat and acidic treatments

To inactivate the lipase, the supernatant was incubated for 5 min at 55°C. After rapid cooling, insoluble materials were removed by centrifugation for 30 min at 12,000 rpm. Afterward, the pH of the previous supernatant was brought to 2.0 by adding 6 N HCl under gentle stirring at 0°C. After centrifugation (30 min at 12,000 rpm), the clear supernatant, which was adjusted to pH 7 with 6 N NaOH, contained 7,800 colipase units per gram of delipidated pancreatic tissue.

#### - Ammonium sulfate precipitation

Extract from stingray pancreas was brought to 50% saturation with solid ammonium sulfate under stirring conditions and maintained for 30 min at 4°C. After centrifugation (30 min, 12,000 rpm), precipitate was resuspended in a minimum volume of extraction solution. Insoluble proteins were discarded by centrifugation (15 min, 12,000 rpm). Preparations of colipase contained of about 80% of the starting amount of colipase.

#### - Ethanol fractionation

Supernatant issued from ammonium sulfate precipitation was subjected to fractionation using ethanol. We added an equal volume of ethanol at 0°C. Insoluble proteins were removed by centrifugation, and the ethanol (4 v/v) was added slowly to the supernatant, bringing the solvent concentration to 90% (v/v) at 0°C. Precipitated proteins, which contained 65% of the starting amount of colipase, were collected and solubilized in minimum volume of 100 mM acetate buffer, pH 4.5, containing 0.05% Triton X-100 and 2 mM benzamidine (buffer A). Insoluble proteins were discarded by centrifugation (10 min, 12,000 rpm) and the clear supernatant thus obtained was dialyzed overnight at 4°C against buffer A.

#### - FPLC anion exchange Mono-S Sepharose

**Figure 4:**
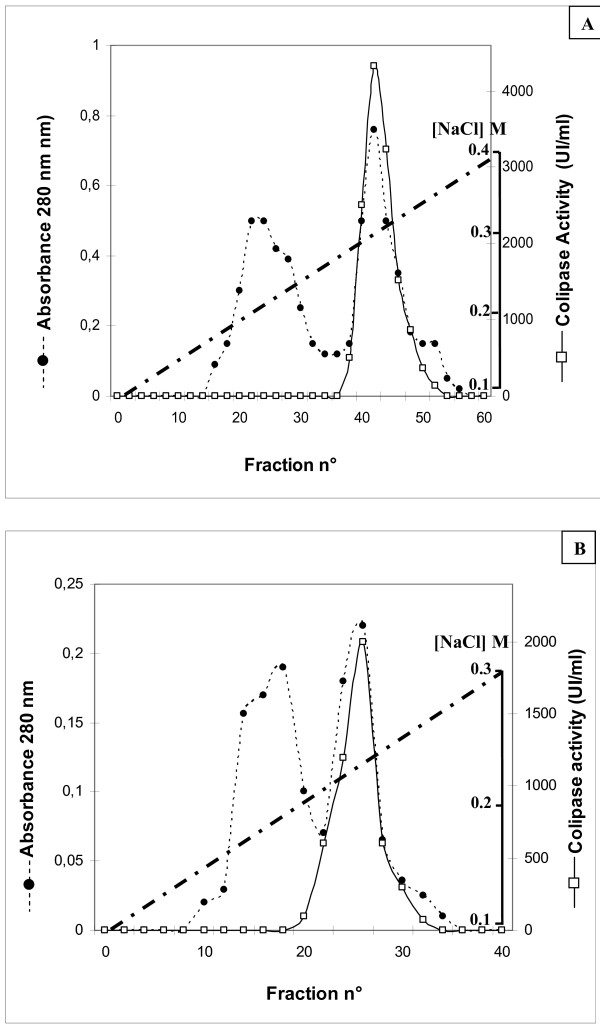
**Chromatography of stingray colipase on FPLC Mono-S Sepharose and FPLC Mono-Q Sepharose**. (A) Chromatography of stingray pancreatic colipase on FPLC Mono-S Sepharose. The column (2.6 cm × 20 cm) was equilibrated with 100 mM acetate buffer, pH 4.5, containing 0.05% Triton X-100 and 2 mM benzamidine (buffer A); a linear salt gradient (0.1 to 0.4 M NaCl) in buffer A was applied to the column; gradient chamber 200 ml; 3 ml fraction; flow rate, 30 ml/h. (B) Chromatography of stingray pancreatic colipase on FPLC Mono Q Sepharose step. The column (2.6 cm × 20 cm) was equilibrated with 10 mM tris-HCl buffer, pH 8, containing 10 mM NaCl and 2 mM benzamidine (buffer B); a linear gradient (0.1 to 0.3 M NaCl) in buffer B was applied to the column; 3 ml fraction; flow rate, 30 ml/h.

The colipase sample (10 ml; 45000 UT) was submitted to FPLC Mono-S Sepharose column previously equilibrated with buffer A. The column (2.6 cm × 20 cm) was rinsed with 0.1 M NaCl in buffer A. Then, proteins were eluted with a linear gradient of NaCl prepared in buffer B. CoSPL was eluted at a salt concentration of 280-330 mM NaCl (figure 4A). Active fractions were pooled, lyophilized for the purpose of concentration and then dialyzed overnight against 10 mM tris-HCl buffer, pH 8, containing 10 mM NaCl and 2 mM benzamidine (buffer B).

#### - FPLC Anion exchange chromatography

Dialyzed active fractions were subjected to anion-exchange chromatography using a Mono Q column (2.6 cm × 20 cm) equilibrated with buffer B. Non bound proteins were washed out with 200 ml of buffer B. After a wash with 100 ml of 0.1 M NaCl in buffer B, elution was performed with a linear gradient of NaCl (0.1-0.3 M). Stingray colipase activity emerged in a single peak at a NaCl concentration of 200 mM (figure 4B). The recovery of colipase activity after the Mono Q step was 75%. Colipase was lyophilized and conserved at -20°C.

## Conclusion

In recent years, characterizations of enzymes from aquatic species have taken place and this had led to the emergence of new applications of these enzymes. In this study, we provided evidence that marine pancreas contains cofactors with very similar biochemical properties with those of turkey, chicken and dromedary cofactors, despite differences observed in the affinity among marine, bird and mammal colipases. Thus, marine, bird and mammal pancreatic systems appear to be functionally similar. The fact that colipase is a universal lipase cofactor might thus be explained by a conservation of the colipase-lipase interaction site. The results obtained in the study may improve our knowledge of marine lipase/colipase. In fact, the lipase/colipase system could be an interesting target to improve the control of the industrial processing of seafood during handling, chilled and frozen storage.

## Competing interests

The authors declare that they have no competing interests.

## Authors' contributions

ABB and AK carried out all the studies, analyzed the data and drafted the manuscript. LD, EB and MB helped in the purification experiments. YG helped with the discussion of the data and the correction of the manuscript. YBA participated in the study design and helped to draft the manuscript. All authors have read and approved the final manuscript.
